# Association of Obesity, Multiple Chronic Conditions, and Frailty: the National Health and Aging Trends Survey

**DOI:** 10.1093/geroni/igab046.2337

**Published:** 2021-12-17

**Authors:** John Batsis, Christian Haudenschild, Anna Kahkoska, rebecca Crow, David Lynch, Curtis Petersen, Matthew Lohman

**Affiliations:** 1 University of North Carolina at Chapel Hill, Chapel Hill, North Carolina, United States; 2 University of Minnesota, University of Minnesota, Minnesota, United States; 3 University of North Carolina at Chapel Hill, University of North Carolina at Chapel Hill/Chapel Hill, North Carolina, United States; 4 VA White River Junction, White River Junction, Vermont, United States; 5 University of North Carolina, Chapel Hill, North Carolina, United States; 6 Dartmouth College, Lebanon, New Hampshire, United States; 7 University of South Carolina, Columbia, South Carolina, United States

## Abstract

As life expectancy increases, so does the risk of developing multiple chronic conditions (MCC). This is concerning as there is a growing obesity epidemic in older adults which is also associated with developing chronic diseases. Both obesity and MCC also increase the risk of frailty, yet the intersection of the three is not well understood. We evaluated the relationship between obesity, multimorbidity, and frailty using data from adults ≥65 years from the National Health and Aging Trends Survey. Obesity was classified using standard body mass index categories (e.g., ≥30kg/m2) and waist circumference (WC; females≥88cm; males≥102cm). MCC was classified as having ≥2 chronic conditions. Adjusted logistic regression models evaluated the association of BMI or WC categories on MCC (yes/no). An analysis limited to persons with obesity evaluated the relationship between frailty phenotypes (e.g, robust, pre-frail, frail) and MCC. Of the 4,967 participants (59.7% female), 79% resided in a private residence. The 70-79 age category was most prevalent. In those with MCC, there were 1,511 (30.4%) classified as having obesity using BMI, and 3,358 (67.6%) using WC. In those without MCC, there were 287 (17.6%) and 744 (51.7%). Compared to normal BMI, the odds of MCC was 0.71 [0.46,1.09], 1.25 [1.08,1.45] and 2.59 [2.15,3.11] in underweight, overweight and obesity. In pre-frailty and frailty, the odds of MCC were 2.52 [1.77,3.59] and 8.35 [3.7,18.85] in BMI-defined obesity. Using WC, the odds were 2.38 [1.94,2.91], and 5.89 [3.83,9.06]. Obesity using both BMI and WC are both strongly associated with multimorbidity and frailty.

